# Seasonal influenza vaccine policy, use and effectiveness in the tropics and subtropics – a systematic literature review

**DOI:** 10.1111/irv.12374

**Published:** 2016-05-26

**Authors:** Siddhivinayak Hirve, Philipp Lambach, John Paget, Katelijn Vandemaele, Julia Fitzner, Wenqing Zhang

**Affiliations:** ^1^ Global Influenza ProgramWorld Health OrganizationGenevaSwitzerland; ^2^ Initiative for Vaccine ResearchWorld Health OrganizationGenevaSwitzerland; ^3^ NIVELUtrechtThe Netherlands

**Keywords:** Influenza vaccines, policy, treatment effectiveness

## Abstract

**Aim:**

The evidence needed for tropical countries to take informed decisions on influenza vaccination is scarce. This article reviews policy, availability, use and effectiveness of seasonal influenza vaccine in tropical and subtropical countries.

**Method:**

Global health databases were searched in three thematic areas – policy, availability and protective benefits in the context of human seasonal influenza vaccine in the tropics and subtropics. We excluded studies on monovalent pandemic influenza vaccine, vaccine safety, immunogenicity and uptake, and disease burden.

**Results:**

Seventy‐four countries in the tropics and subtropics representing 60% of the world's population did not have a national vaccination policy against seasonal influenza. Thirty‐eight countries used the Northern Hemisphere and 21 countries the Southern Hemisphere formulation. Forty‐six countries targeted children and 57 targeted the elderly; though, the age cut‐offs varied. Influenza vaccine supply increased twofold in recent years. However, coverage remained lower than five per 1000 population. Vaccine protection against laboratory‐confirmed influenza in the tropics ranged from 0% to 42% in the elderly, 20–77% in children and 50–59% in healthy adults. Vaccinating pregnant women against seasonal influenza prevented laboratory‐confirmed influenza in both mothers (50%) and their infants <6 months (49–63%).

**Conclusion:**

Guidelines on vaccine composition, priority risk groups and vaccine availability varied widely. The evidence on vaccine protection was scarce. Countries in the tropics and subtropics need to strengthen and expand their evidence‐base required for making informed decisions on influenza vaccine introduction and expansion, and how much benefit to expect.

## Introduction

Influenza may affect up to 10% of the world's population every year 1 with substantial morbidity and mortality.^2^, ^3^, ^4^, ^5^ With immunization being one of the most powerful and cost effective interventions against infectious diseases,^6^ vaccination remains the main strategy to protect populations against influenza‐associated morbidity and mortality. The World Health Assembly (WHA 56.19) resolved in 2003 to increase the use of seasonal influenza vaccines to protect individuals at high risk for influenza and related complications,^7^ and in 2005, it mandated the WHO (WHA58.5) to work with international and national partners to increase access to influenza vaccines.^8^ The WHO Global Action Plan for Influenza Vaccines launched in 2006 aims to promote evidence‐based use of seasonal influenza vaccine as a strategy towards pandemic preparedness.^9^, ^10^ The immediate goal is to increase by 2016 the global vaccine production capacity to produce enough vaccine to equitably immunize 70% of the world population with a pandemic vaccine that gives an adequate protection within 6 months of vaccine seed transfer to manufacturers in the event of a pandemic.^11^

Over the last decade, an increasing number of countries in the tropics and subtropics have introduced seasonal influenza vaccination in their national immunization policies and/or expanded their policies to include maternal influenza immunization.^12^, ^13^ This is critical given the WHO Strategic Advisory Committee of Experts on Immunization recommendation in 2012 that countries with seasonal influenza vaccine programmes prioritize persons at high risk of severe influenza virus infection, including pregnant women at any stage of their pregnancy; children aged 6 months to 5 years, the elderly and individuals with underlying health conditions such as HIV/AIDS, asthma and chronic heart or lung diseases.^14^ Unlike in the temperate regions, influenza seasonality in the tropics is less distinct with multiple and less pronounced peaks with often year‐round transmission.^15^, ^16^ In the absence of clear guidance on which vaccine formulation to use and when to vaccinate in the tropics and subtropics, countries often lack evidence to make informed decisions. As one of the early steps of a larger WHO effort to improve seasonal influenza vaccine use and pandemic preparedness in the tropics and subtropics, this article reviews the current status of (i) national policy and guidelines, (ii) coverage and use and (iii) efficacy and effectiveness, of seasonal influenza vaccine in the context of the tropics and subtropics.

## Methods

The Preferred Reporting Items for Systematic Reviews and Meta‐analysis (PRISMA) statement was used to guide the process and reporting of our review (Appendix A).^17^The outcomes of interest were to review the current status of national policy for seasonal influenza vaccination, national guidelines for priority population groups and vaccine composition, vaccine use and effectiveness. Vaccination coverage was estimated as the number of vaccine doses distributed expressed per 1000 population. Vaccine effectiveness estimates were reviewed separately for laboratory‐confirmed influenza, for different risk groups, further stratified by type of vaccine and antigenic match wherever possible.

We searched multiple databases (United States National Library of Medicine, Cochrane Library, WHO Library Information System, Latin American and Caribbean Health Sciences Literature, National Databases of Indian Medical Journals) using different combinations of search terms (with synonyms and closely related words) such as ‘seasonal influenza’, ‘influenza vaccine’, ‘tropics’, ‘effectiveness’, ‘efficacy’, ‘policy’, ‘guidelines’, ‘timing’, ‘composition’, ‘coverage’, ‘availability’, ‘uptake’, ‘production’, ‘manufacturing’ and ‘campaign’. An example of a search query is shown in Appendix B. We screened the title and abstract of all articles to determine eligibility. We also screened other articles that showed up as related during the search. We retrieved the full text of all eligible articles and further assessed for inclusion. We screened articles that were newly identified in the references cited in the full‐text articles. We reviewed all the past systematic reviews and meta‐analyses on vaccine efficacy and effectiveness as well as all the individual studies that had been included in these reviews. Duplicates were removed and the title and abstract were screened for eligibility by two reviewers independently. Articles were included based on consensus between the two reviewers. For multiple articles referring to the same study, we included the article with the most recent findings. To access grey literature, we first contacted institutions, networks and individuals known to be involved in influenza research, used snow‐balling technique to further identify other influenza researchers and searched conference proceedings and agency reports to identify ongoing or unpublished studies. Researchers were requested to share preliminary summaries of unpublished studies to assess their eligibility for inclusion in the review. We plotted funnel plots of estimates of vaccine effectiveness and efficacy for published and unpublished studies separately for elderly, children and healthy adults to assess the extent of publication bias if any. We also reviewed four global databases on seasonal influenza vaccine use administered by the WHO and UNICEF or maintained by vaccine manufacturers. We triangulated information regarding influenza policy, guidelines and coverage from the different global databases – in case of discordant findings, we considered the information from the most recent database as valid.

We limited our review to 138 countries and territories (excluding Australia) representing 79% of the world's population, situated partly or wholly between the north and south 38th lateral (Appendix C) and restricted our inclusions to articles in the English language or any other language provided an abstract was available in English. We included articles related to policies and guidelines for seasonal human influenza vaccine use in tropical and subtropical countries, articles that referred to seasonal influenza vaccine composition, timing of vaccination, vaccine production, availability and coverage, and vaccine efficacy and effectiveness. We excluded studies that focused on avian or pandemic influenza vaccine or pandemic preparedness. Studies on safety and immunogenicity of influenza vaccines, determinants of influenza vaccine uptake, licensing and regulatory aspects of influenza vaccine were also excluded. Studies that focused solely on influenza seasonality, disease burden, antigenic and genetic characteristics were beyond the scope of this review. Data was extracted directly from the full‐text articles into structured tables containing all the relevant variables of interest such as the status of influenza vaccination policy, year in which vaccine policy was introduced, vaccine formulation used, targeted high‐risk population groups, whether available in the private or public sector, vaccine coverage, etc. For vaccine effectiveness and efficacy studies, we extracted information on the study year, study design, study population (size and risk group types), vaccine type (live attenuated or inactive), formulation used (Northern or Southern Hemisphere), ascertainment of exposure (i.e. vaccination), outcome (e.g. influenza‐like illness, pneumonia, hospitalization, laboratory‐confirmed influenza), efficacy, effectiveness and other potential confounding factors such as antigenic match of vaccine with circulating influenza virus. Simple proportions with 95% confidence intervals, the range of proportions across different studies, stratified by relevant variables such as age were used to describe the outcome variables. We did not repeat a meta‐analysis for vaccine efficacy and vaccine effectiveness, as more than 20 such efforts (including 11 Cochrane Collaboration reviews) have been carried out in the past. Moreover, we did not identify any new published studies from the tropics and subtropics since the last meta‐analysis of studies from low‐ and middle‐income countries.^18^ The risk of bias in the selection and ascertainment of exposure and outcome for cohort and case–control vaccine effectiveness studies was assessed independently by two reviewers (SH and JS) using the Newcastle–Ottawa bias assessment scale.^19^ We similarly assessed the methodological quality for the vaccine efficacy trials based on randomization, allocation, blinding and follow‐up using the Cochrane risk of bias assessment tool.^20^ Wherever appropriate, we triangulated information from the global databases to validate the information extracted from the literature.

## Results

**Figure 1:**
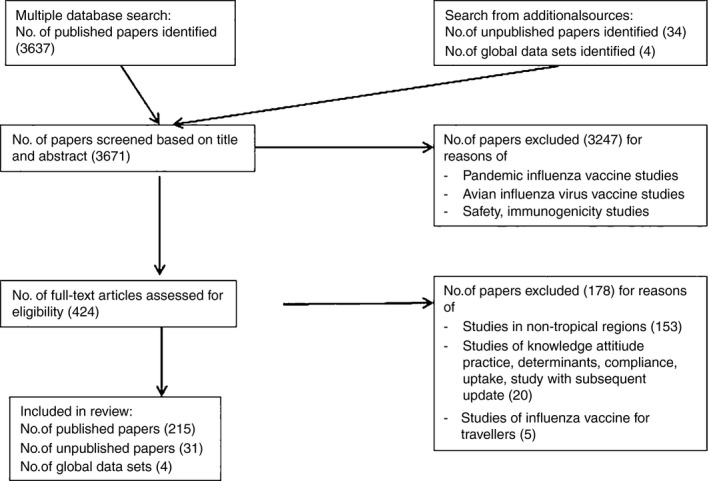
PRISMA flowchart of selection and exclusion of articles.

Of the 3637 articles and 34 unpublished papers identified as of 30 September 2014, 3247 were deemed ineligible based on the screening of the title and abstract. A further 178 articles were excluded after a full‐text appraisal. A total of 215 published (vaccine policy and guidelines – 27, timing and composition – 49, supply, availability and coverage – 37 and vaccine efficacy – 102) and 31 unpublished articles (vaccine policy and guidelines – 8, timing and composition – 4, supply, availability and coverage – 8 and vaccine efficacy – 11) and four global databases on seasonal influenza vaccine use were included in the final review (Figure 1). The risk of bias in selection of the vaccinated cohort, comparability of the vaccinated and non‐vaccinated participants, outcome ascertainment or differential loss to follow‐up was high in most cohort studies (Appendix D). The risk of bias in selection of cases or dissimilar non‐response rate in cases and controls was high in most case–control studies (Appendix E). The risk of bias was lower (except in three non‐randomized controlled trials) in the vaccine efficacy trials (Appendix F). Most of the published systematic reviews and meta‐analysis (especially the reviews published after 2000) referred to the Cochrane Manual for systematic reviews; though, only one review explicitly stated that it adhered to the PRISMA statement. There was no evidence to suggest publication bias in the context of vaccine effectiveness studies (results not shown). The review covers 138 countries and territories representing about 79% of the world's population situated in the tropics and subtropics.

### Seasonal influenza immunization policy

**Figure 2:**
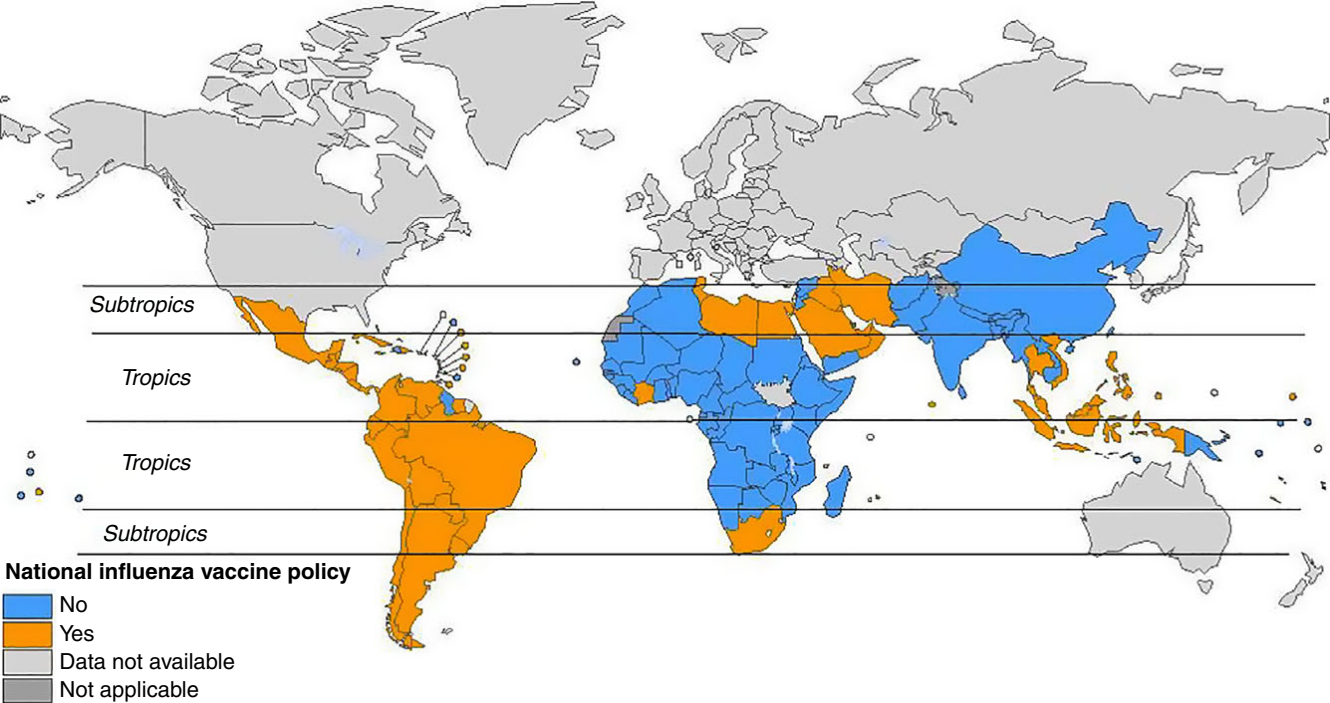
Countries in the tropics and subtropics with a national influenza immunization policy.

Sixty‐four of 138 tropical and subtropical countries representing 20% of the world's population had a national vaccination policy against seasonal influenza (Figure 2).^21^ Countries such as Bangladesh, China, India, Pakistan and Sri Lanka in Asia representing about 45% of the world's population did not have a national vaccination policy against influenza. All of Central and South America except Guyana, Haiti, Saint Kitts & Nevis and Saint Vincent and the Grenadines had a seasonal influenza immunization policy.^22^ On the other hand, only six countries (Côte d'Ivoire, Egypt, Libya, Mauritius, Tunisia and South Africa) in Africa had a national immunization policy or guideline against seasonal influenza.

**Figure 3:**
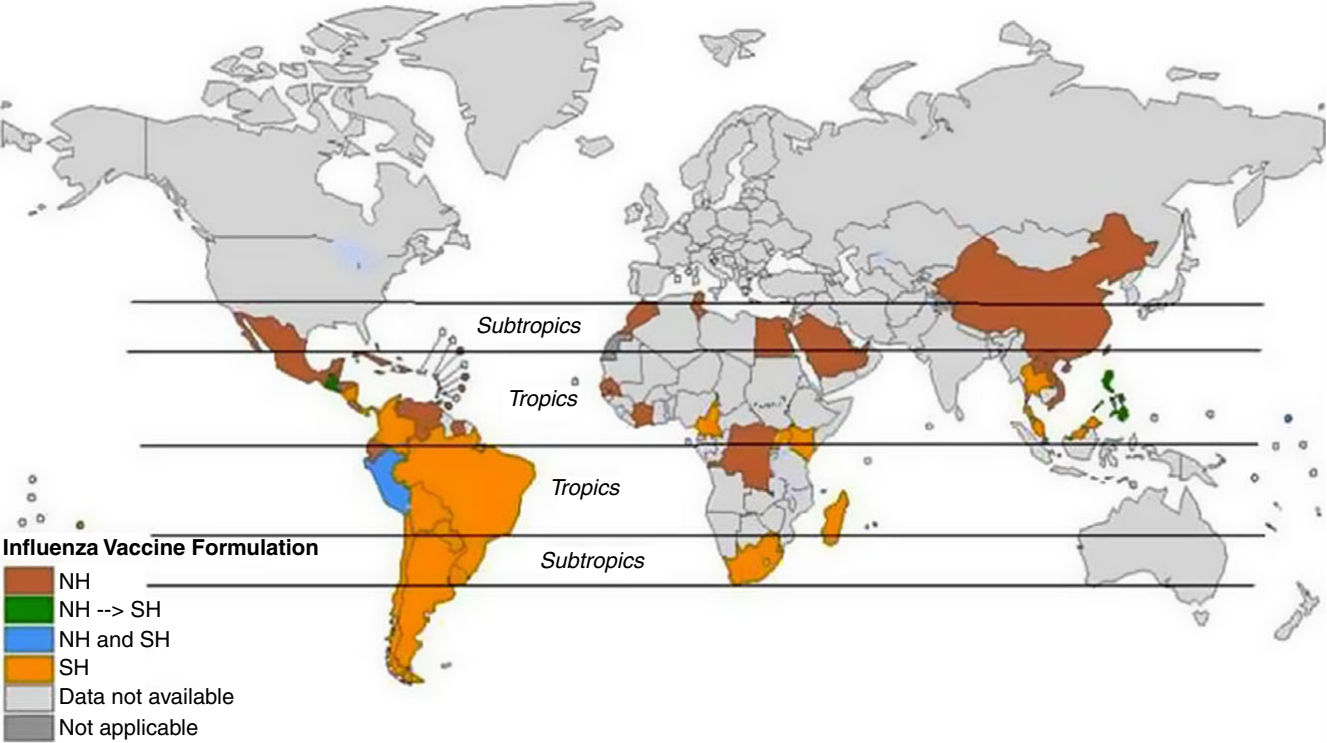
National guidelines on seasonal influenza vaccine formulation for countries in the tropics and subtropics. NH, Northern Hemisphere; SH, Southern Hemisphere.

National policy guidance on vaccine composition was available for 63 countries – 38 and 21 countries recommended the use of the Northern and Southern Hemisphere formulation respectively, whereas three countries (Brunei Darussalam, Marshall Islands and Singapore) recommended both formulations. Peru experimented with both formulations in 2012 but subsequently reverted to one formulation. Five countries situated in the Southern Hemisphere tropics used the Northern Hemisphere formulation, whereas eight situated in the Northern Hemisphere tropics used the Southern Hemisphere formulation. Moreover, four countries (Costa Rica, El Salvador, Guatemala and the Philippines) situated in the Northern Hemisphere tropics had switched from a Northern to a Southern Hemisphere formulation in recent years (Figure 3).

Countries with a national policy for influenza vaccine followed the WHO recommendations for targeting one or more high‐risk groups – the elderly, children, individuals with underlying health conditions such as HIV/AIDS, asthma and chronic heart or lung diseases and healthcare professionals.^14^ The age range of children and the elderly targeted for influenza immunization varied among the countries.^23^ Forty‐six countries targeted children of 6 months to 5 years, whereas 57 countries targeted the elderly either above 60 or 65 years of age.^21^ Sixty‐one countries targeted individuals with underlying health conditions such as HIV/AIDS, asthma and chronic heart or lung diseases. Forty‐seven countries (including 27 in Central and South America) recommended seasonal influenza immunization during pregnancy. Fifty‐one countries recommended vaccination of healthcare professionals against influenza. There was no correlation between the development status of the country and free influenza vaccination to healthcare professionals.^24^, ^25^ A survey in 2009 showed that 32 of the 35 countries in Central and South America administered influenza vaccines to their healthcare professionals through the public health systems.^26^ Healthcare professionals were more commonly targeted for vaccination after the 2009 pandemic.^25^ In Saudi Arabia, the vaccine was mandatory for all health professionals working in the Hajj pilgrimage centres of Mecca and Medina. Saudi Arabia also required all incoming Hajj pilgrims to be vaccinated against influenza.^27^, ^28^

### Vaccine supply, availability and coverage

Vaccine coverage varied widely across countries (Appendix G). Seasonal influenza vaccine supply increased from 350 million doses in 2006 to around 900 million in 2009.^29^ It increased by 87% between 2004 and 2011 but by only 3% per annum in the last 3 years.^30^ Less than 20% of the global seasonal influenza vaccine was produced by regional manufacturers in tropical and subtropical countries.^31^ In China, despite an 18% annual increase in vaccine supply since 2005, local manufacturers supplied 32·5 million doses of seasonal influenza vaccine in 2008–2009 season against an estimated domestic need of 570 million doses per year.^32^ Fourteen manufacturers from the tropical and subtropical regions received technology transfer support that was expected to enhance the supply to 795 million doses by 2016.^33^, ^34^, ^35^, ^36^ Vaccine manufacturers in India, Indonesia and Thailand had since started production of seasonal influenza vaccine. Mexico, South Africa, Egypt, the Islamic Republic of Iran, Thailand and Viet Nam were expected to begin production within 5–10 years.^37^ There was no production capacity in sub‐Saharan Africa except one facility in South Africa that filled and packaged imported vaccine.^38^

Influenza vaccine was available through the public sector in 50 countries. In another 28 countries, it was available solely through the private sector. However, usage was low when the vaccine was available only in the private sector.^30^ Influenza vaccine was available in 14 of the 31 countries surveyed in Africa – six through the private sector (Democratic Republic of the Congo, Senegal, Togo, Uganda, Zambia and Zimbabwe) and the remaining eight through both public and private sectors (Cameroon, Côte d'Ivoire, Egypt, Kenya, Madagascar, Mauritius, Morocco and South Africa).^39^

**Figure 4:**
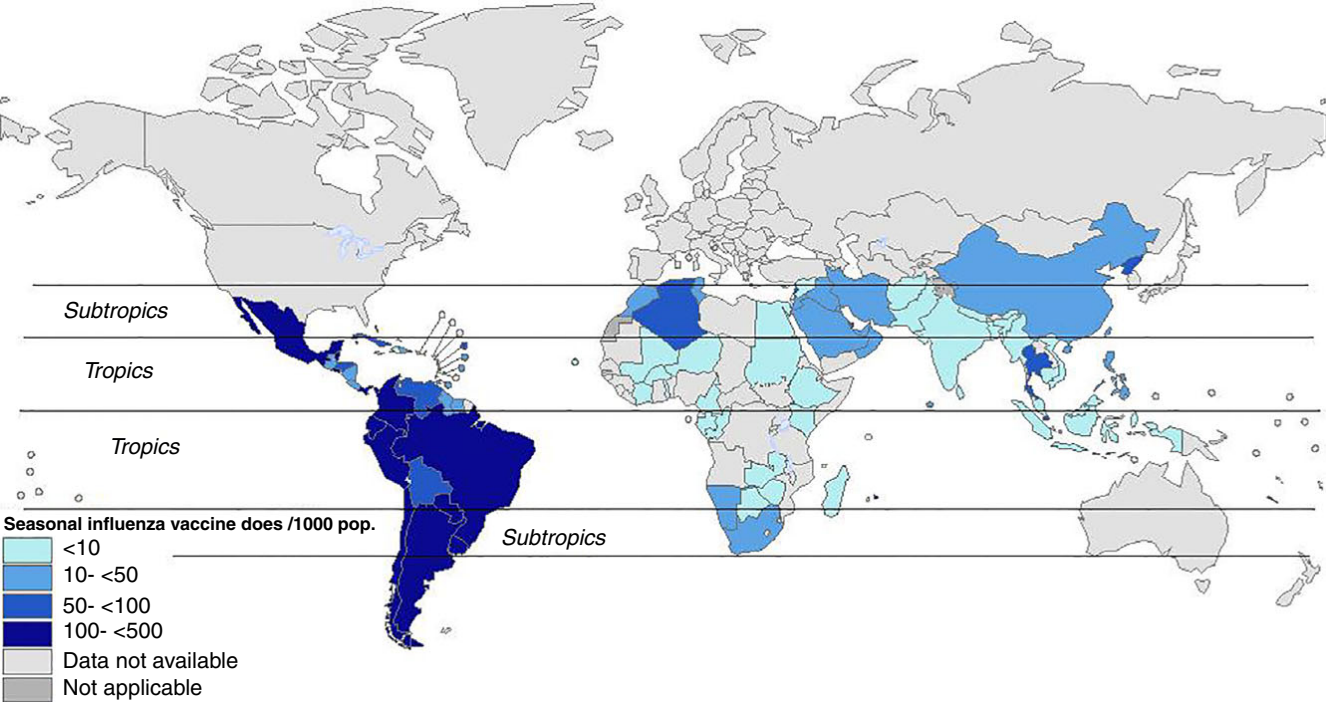
Seasonal influenza vaccine doses distributed in the tropics and subtropics (2011) (Source: adapted from Ref. 32).

Despite a twofold increase in recent years, influenza vaccine coverage was <5 per 1000 general population in tropical and subtropical countries (Figure 4).^40^ The greatest increase in coverage (2008–2011) was seen in Asia. However, the total number of seasonal influenza vaccine doses distributed was relatively small at 8·2 million in 2011. Coverage, estimated as the proportion of seasonal influenza vaccine doses distributed in the general population, was <1% in 2011 in all of Africa (except Algeria, Mauritius, Morocco, Namibia, Tunisia and South Africa) 39 and Asia (Bangladesh, India, Indonesia, Myanmar, Nepal and Sri Lanka). The coverage was 7–12 per 1000 in Singapore despite influenza vaccines being offered at a cost in public hospitals.^41^ In contrast, countries in Central and South America (except Guatemala, Guyana, Haiti and Jamaica), Mauritius and China, Hong Kong SAR reported a coverage of more than one dose per 1000 population.^30^ Seasonal influenza vaccination coverage varied widely across different targeted population groups (Table 1) in the tropics.

**Table 1:** Range of estimates for seasonal influenza vaccine coverage in the tropics and subtropics

Seasonal influenza vaccine coverage	Period	Countries	References
Children	2014	Argentina, Belize, Chile, Colombia, Ecuador, El Salvador, Mexico, Nicaragua, Panama: >80%; Bolivia (Plurinational State of), Honduras, Paraguay, Peru, Uruguay: 23–45%	76
	2006	Public funded (Chile, China – Province of Taiwan): 23–62%, User paid (Argentina): 8–10%	77
	2011–2012	China – Province of Taiwan: 32–72%	78
	2010–2012	Thailand: <2%	79
Elderly	1993–1997 2004–2008 2014	Chile, Costa Rica, Dominican Republic, El Salvador, Honduras, Mexico and Nicaragua: >75%; Argentina, Belize, Bolivia (Plurinational State of), Colombia, Ecuador, Panama, Paraguay, Peru and Uruguay: 20–69%	26, 76, 80
	2000–2003	Brazil: 66–90%	81, 82, 83, 84, 85, 86, 87, 88, 89, 90, 91
	2006	Public funded (Argentina, Chile, Republic of Korea): 14–41%; User paid (China, South Africa): <10%	77
Pregnant women	2005–2006 2010–2013	India, China, Hong Kong SAR, Thailand: 0–4%	79, 92, 93, 94
Healthcare professionals	2001–2005 2012	Brazil, China, 20–56%	95, 96, 97

### Vaccine efficacy and effectiveness

Vaccine efficacy and effectiveness against different outcomes such as laboratory‐confirmed disease, influenza‐like illness, hospitalization and mortality associated with seasonal influenza varied widely in different high‐risk groups in tropical and subtropical countries (Appendix H–L). The 21 studies in the elderly that were reviewed comprised four randomized and two non‐randomized controlled trials, seven cohort studies, four case–controls and four ecological studies. Seasonal influenza vaccination provided 43–58% protection in the elderly.^18^, ^42^, ^43^ The seven studies in healthy adults comprised one randomized controlled trial, and three cohorts and case–control studies each. Seasonal influenza vaccination provided 50–59% protection in healthy adults.^18^, ^44^, ^45^, ^46^ The 12 studies in children comprised nine randomized controlled trials, one cohort and two case–control studies. Seasonal influenza vaccination provided 20–77% in children depending on antigenic match, against laboratory‐confirmed influenza (Table 2).^18^, ^47^, ^48^, ^49^, ^50^, ^51^, ^52^, ^53^, ^54^, ^55^ Vaccinating school children provided 23·3% (66·3–74·9) protection against influenza and indirect protection of 61% (5·8–84·7) for household contacts.^56^ Vaccinating pregnant women prevented laboratory‐confirmed influenza in both mothers (50%) and their infants up to 6 months of age (49–63%).^57^, ^58^ Vaccinating patients with chronic obstructive pulmonary disease provided 70% protection against laboratory‐confirmed influenza in Thailand.^59^

**Table 2:** Range of VE estimates from meta‐analytic reviews and individual studies from the tropics and subtropics

Outcome	Range of pooled VE estimates from meta‐analytic reviews	References	Range of VE point estimates from individual studies from the tropics and subtropics	References
Elderly				
ILI	4–59%	18, 61, 62	0–76%	42, 90, 98, 99, 100, 101
LCI	43–58%	18, 61, 62	0–42%	42, 43
Pneumonia	30–53%	61, 102, 103	43%	104
Hospitalization				
Influenza‐related	No effect – 33%	18, 62, 102, 103, 105, 106, 107	31–77%^a^	43, 80, 104, 108, 109, 110, 111, 112, 113, 114, 115, 116, 117
All‐cause	50%		–	
Mortality				
Influenza‐related	8–30%	61, 62, 102, 103, 106, 107	20–53%^a^	43, 104, 108, 113, 116, 118, 119, 120, 121
All‐cause	36–68%		No effect – 44%	
Children				
ILI	31–45% LAIV: 36% TIV: 27%	18, 63, 67, 70, 71, 72	8–85%	47, 101
LCI				
Overall	67–74%	18, 63, 64, 65, 66, 67, 68, 69, 70, 71, 72, 73	20–77%	47, 48, 49, 50, 51, 52, 53, 54, 55, 114, 122, 123
LAIV	62–83%		64–72%	
TIV	48–72%		33–62%	
LAIV good match	61–88%		70–78%	
TIV good match	48–81%			
LAIV poor match	60–87%			
TIV poor match	49–56%			
One dose	58%		58%	
Two doses	75%		74%^b^	
Influenza A	31–91%		25–57%	
Influenza B	45%		0–50%	
Healthy adults				
ILI				
LAIV	10%	18, 65, 74, 124	–	101, 125, 126, 127
TIV	20–69%		39–73%	
LCI				
TIV good match	57–80%	18, 64, 65, 66, 74, 124		44, 45, 46
TIV poor match	44–52%			
TIV any match	59–82%		50–59%	
TIV influenza A	64%		H1N1pdm – 84%, H3N2 – 33%	
TIV influenza B	52%		84%	
Pregnant women				
ILI in mother				
Healthy mothers	0–44%	128, 129, 130, 131, 132, 133	0–36%	57, 58
HIV‐infected mothers	–		No effect	
LCI in mother				
Healthy mothers	–		50%	58
HIV‐infected mothers	–		58%	
ILI in infant				
Healthy mothers	No effect	128, 129, 130, 131, 132, 133	0–29%	57, 58
HIV‐infected mothers	–		No effect	
LCI in infant				
Healthy mothers	–		49–63%	57, 58
HIV‐infected mothers	–		27% (ns)	
Preterm/IUGR (infant)	0–72%	134	28–37% (ns)	57
High‐risk individuals				
COPD patients				
ARI	11% (ns)	135	60–85%	59, 136, 137
LCI	81%		71%	
Hospitalization (ARI)	67% (ns)		72%	
Coronary heart disease patients				
Coronary heart disease mortality	61%	138	38 (ns) – 66%	139, 140
HIV‐infected patients				
ARI	–	141, 142	8 (ns) – 16% (ns)	143
LCI	27–78%		76%	
Healthcare professionals	No effect on LCI, hospitalization or mortality in elderly who received care from healthcare professionals	144	51% against ILI in health workers if good antigenic match; no effect if poor antigenic match	145
Pilgrims				
ILI	72%	18	38–77%	146, 147

ARI, acute respiratory illness; COPD, chronic obstructive pulmonary disease; HIV, human immunodeficiency virus; ILI, influenza‐like illness; LAIV, live attenuated influenza vaccine; LCI, laboratory‐confirmed influenza; TIV, trivalent inactivated influenza vaccine; VE, vaccine efficacy and effectiveness.

a No effect in two studies.

b One study showed protective effect only after two doses.

## Discussion

This paper presents a comprehensive review of influenza policy, availability, coverage, use and effectiveness of seasonal influenza vaccine currently in the tropics and subtropics. Most countries in Europe, United States and the developed world have a national policy on immunization against seasonal influenza.^21^, ^60^ In contrast, large parts of sub‐Saharan Africa and the Indian subcontinent are yet to formulate national policies against seasonal influenza; though, the vaccine is available through the private sector in many countries in the region. Historically, the Central and South Americas have often led the introduction of new and underused vaccines including expansion of seasonal influenza vaccine to pregnant women. Even as some countries such as the United States expand their policy to immunize all individuals above 6 months of age against influenza, targeted vaccination of high‐risk population subgroups remains the main strategy to reduce influenza disease burden. Many countries in the tropics recommended the Northern or Southern Hemisphere vaccine formulation based on geographical location rather than on evidence on local seasonality and antigenic evolution patterns.

Overall, seasonal influenza vaccination coverage was <1% in most parts of Africa and Asia. In contrast, reported coverage in the Central and South America was comparable to that seen in high‐income countries.^30^ Higher vaccination coverage is not correlated with higher level of economic development. Higher coverage, however, is seen when the vaccine is offered at no cost through the public sector.^12^ Estimating influenza vaccine coverage poses major challenges for countries in the tropics and subtropics – lack of special vaccine coverage studies, suboptimal definition of coverage, absence of reliable denominators for the targeted high‐risk population groups, lack of information on unused or returned or wasted vaccine doses, among others. Vaccine coverage estimated by national immunization programmes is less meaningful as they are based on the number of vaccine doses distributed or administered expressed as a fraction of number of doses procured. Global distribution of vaccines by the major manufacturers has been used as a proxy for vaccine coverage.^30^ However, these coverage estimates do not account for 21% of the global vaccine supply by developing country manufacturers that do not contribute to this database.

Our review suggests that the 43–58% protection by the vaccine against laboratory‐confirmed influenza in the elderly was slightly lower in the tropics and subtropics compared to 50–77% protection seen in Europe, United States and other developed countries.^61^, ^62^ The protection in children (range 20–77%) and in healthy adults (range 50–59%) seen in the tropics and subtropics was comparable with that seen in developed countries.^63^, ^64^, ^65^, ^66^, ^67^, ^68^, ^69^, ^70^, ^71^, ^72^, ^73^, ^74^ Vaccine protection studies from the tropics and subtropics faced certain limitations. First, the majority of the studies from the tropics and subtropics were observational and prone to selection or ascertainment bias. The outcome for assessing vaccine protection was based on clinical ascertainment or self‐reports without laboratory confirmation of influenza. Three of 16 vaccine efficacy trials were non‐randomized and prone to ascertainment and reporting bias. Second, most studies were from large urban areas with limited national representativeness. Third, few studies matched for antigenic relatedness of the circulating influenza virus with the vaccine virus strain. Vaccine effectiveness is known to be low during seasons of antigenic mismatch as was seen during the 2014–2015 influenza season in the United States when protection was much lower [18% (95% CI: 6–29%)] against A(H3N2) viruses.^75^

Notwithstanding these challenges, vaccine effectiveness data is critical for countries to introduce and evaluate influenza vaccination programmes. Even as an increasing number of countries from the tropics and subtropics introduce influenza into their immunization programme, there are critical knowledge gaps in these settings. It is not known how the high prevalence of underlying malnutrition and infections such as tuberculosis, malaria and limited access to health care may increase the risk of influenza‐related complications or may influence vaccine effectiveness. Although children and the elderly are at high risk of severe influenza outcomes, few studies from the tropics and subtropics assessed severe outcomes such as influenza‐related hospitalization or mortality. Almost a third of these studies were observational and prone to bias due to self‐assessment of the influenza outcomes. Only a few studies evaluated vaccine effectiveness stratified by antigenic match or reported data on vaccine coverage, influenza incidence and seasonality.^64^ Finally, waning immunity in the months following vaccination may decrease vaccine benefits in tropical and subtropical regions where influenza activity prevails all year round. Countries need to strengthen their national surveillance for influenza that integrate methodologies to measure vaccine coverage and evaluate vaccine effectiveness using well‐defined denominators.

Our review, though systematic, was subject to several methodological and substantive limitations. *First*, we excluded articles in languages other than English. However, we took efforts to search regional databases for articles in Spanish language as countries from Central and South America have contributed substantially to influenza research in the recent past. An earlier review of influenza (seasonal and pandemic) vaccine effectiveness in low‐ and middle‐income countries identified 132 articles of a total of 361, in languages other than English.^18^ More than 75% of these non‐English language articles were from Russia and another 15 articles were from Romania, countries that were outside the scope of our review. Nevertheless, our review may be limited to some extent by the exclusion of potential studies from China.

*Second*, there was wide disparity in the geographic distribution of studies. For example, of the 26 studies from the tropics and subtropics of vaccine effectiveness among the elderly, 11 (including nine from Brazil,) were from Central and South America, 12 from Asia Pacific (including four each from China and Hong Kong SAR, three from Thailand and two from China – Province of Taiwan), and three from Africa (all from South Africa). Most of sub‐Saharan Africa, the Middle East, Bangladesh, India, Indonesia, Pakistan and Sri Lanka in Asia were underrepresented in the review.

## Conclusion

Seventy‐four countries in the tropics and subtropics representing 60% of the world's population did not have a national vaccination policy against seasonal influenza. Guidelines on vaccine composition, priority risk groups and vaccine availability in the public and private sector varied widely. Influenza vaccination coverage was <5 per 1000 general population. The evidence on vaccine effectiveness in the tropics and subtropics was scarce. Countries in the tropics and subtropics need to strengthen and expand their local and regional evidence‐base required for making informed decisions on influenza vaccine introduction and expansion, and how much benefit to expect.

## Supporting information


**Appendix S1.** PRISMA 2009 checklist.
**Appendix S2.** Strategies and keywords used for literature search.
**Appendix S3.** List of countries and territories in the tropics and subtropics included in the review.
**Appendix S4.** Potential risk of bias (shaded in grey) in cohort vaccine effectiveness studies. Risk of bias assessed using Newcastle‐Ottawa Scale.
**Appendix S5.** Potential risk of bias (shaded in grey) in case control vaccine effectiveness studies – Risk of bias assessed using Newcastle‐Ottawa Scale.
**Appendix S6.** Potential risk of bias in randomized controlled trials for vaccine efficacy studies – Risk of bias assessed using Cochrance risk of bias assessment tool. High risk of potential bias or lack of information to assess risk of bias is shaded in grey.
**Appendix S7.** Seasonal influenza vaccine coverage in the tropics and subtropics.
**Appendix S8.** Seasonal influenza effectiveness in the elderly.
**Appendix S9.** Seasonal influenza effectiveness in children.
**Appendix S10.** Seasonal influenza vaccine effectiveness in healthy adults.
**Appendix S11.** Seasonal influenza vaccine effectiveness in pregnant women.
**Appendix S12.** Seasonal influenza vaccine effectiveness in high risk individuals.
