# Supplementary material for: Seasonal influenza vaccine policy, use and effectiveness in the tropics and subtropics – a systematic literature review
Source: Influenza Other Respir Viruses. 2016 May 26;10(4):254–67. doi: 10.1111/irv.12374 (PMC4910173; doi:10.1111/irv.12374)
Supplement: Supplementary file 1 — Appendix S1. PRISMA 2009 checklist. Appendix S2. Strategies and keywords used for literature search. Appendix S3. List of countries and territories in the tropics and subtropics included in the review. Appendix S4. Potential risk of bias (shaded in grey) in cohort vaccine effectiveness studies. Risk of bias assessed using Newcastle‐Ottawa Scale. Appendix S5. Potential risk of bias (shaded in grey) in case control vaccine effectiveness studies – Risk of bias assessed using Newcastle‐Ottawa Scale. Appendix S6. Potential risk of bias in randomized controlled trials for vaccine efficacy studies – Risk of bias assessed using Cochrance risk of bias assessment tool. High risk of potential bias or lack of information to assess risk of bias is shaded in grey. Appendix S7. Seasonal influenza vaccine coverage in the tropics and subtropics. Appendix S8. Seasonal influenza effectiveness in the elderly. Appendix S9. Seasonal influenza effectiveness in children. Appendix S10. Seasonal influenza vaccine effectiveness in healthy adults. Appendix S11. Seasonal influenza vaccine effectiveness in pregnant women. Appendix S12. Seasonal influenza vaccine effectiveness in high risk individuals. [file IRV-10-254-s001.pdf]

Appendix A: PRISMA 2009 checklist

Section/topic	#	Checklist item	Reported on page #
**TITLE**			
Title	1	Identify the report as a systematic review, meta-analysis, or both.	1
**ABSTRACT**			
Structured summary	2	Provide a structured summary including, as applicable: background; objectives; data sources; study eligibility criteria, participants, and interventions; study appraisal and synthesis methods; results; limitations; conclusions and implications of key findings; systematic review registration number.	2
**INTRODUCTION**			
Rationale	3	Describe the rationale for the review in the context of what is already known.	4
Objectives	4	Provide an explicit statement of questions being addressed with reference to participants, interventions, comparisons, outcomes, and study design (PICOS).	5
**METHODS**			
Protocol and registration	5	Indicate if a review protocol exists, if and where it can be accessed (e.g., Web address), and, if available, provide registration information including registration number.	----
Eligibility criteria	6	Specify study characteristics (e.g., PICOS, length of follow-up) and report characteristics (e.g., years considered, language, publication status) used as criteria for eligibility, giving rationale.	6
Information sources	7	Describe all information sources (e.g., databases with dates of coverage, contact with study authors to identify additional studies) in the search and date last searched.	5
Search	8	Present full electronic search strategy for at least one database, including any limits used, such that it could be repeated.	Appendix B
Study selection	9	State the process for selecting studies (i.e., screening, eligibility, included in systematic review, and, if applicable, included in the meta-analysis).	6
Data collection process	10	Describe method of data extraction from reports (e.g., piloted forms, independently, in duplicate) and any processes for obtaining and confirming data from investigators.	7
Data items	11	List and define all variables for which data were sought (e.g., PICOS, funding sources) and any assumptions and simplifications made.	7

Risk of bias in individual studies	12	Describe methods used for assessing risk of bias of individual studies (including specification of whether this was done at the study or outcome level), and how this information is to be used in any data synthesis.	7
Summary measures	13	State the principal summary measures (e.g., risk ratio, difference in means).	5
Synthesis of results	14	Describe the methods of handling data and combining results of studies, if done, including measures of consistency (e.g., I^2) for each meta-analysis.	----
Risk of bias across studies	15	Specify any assessment of risk of bias that may affect the cumulative evidence (e.g., publication bias, selective reporting within studies).	8
Additional analyses	16	Describe methods of additional analyses (e.g., sensitivity or subgroup analyses, meta-regression), if done, indicating which were pre-specified.	----
**RESULTS**			
Study selection	17	Give numbers of studies screened, assessed for eligibility, and included in the review, with reasons for exclusions at each stage, ideally with a flow diagram.	7, figure 1
Study characteristics	18	For each study, present characteristics for which data were extracted (e.g., study size, PICOS, follow-up period) and provide the citations.	Appendix G to L
Risk of bias within studies	19	Present data on risk of bias of each study and, if available, any outcome level assessment (see item 12).	Appendix D to F
Results of individual studies	20	For all outcomes considered (benefits or harms), present, for each study: (a) simple summary data for each intervention group (b) effect estimates and confidence intervals, ideally with a forest plot.	Table 1, 2<br>Appendix G to L
Synthesis of results	21	Present results of each meta-analysis done, including confidence intervals and measures of consistency.	-----
Risk of bias across studies	22	Present results of any assessment of risk of bias across studies (see Item 15).	-----
Additional analysis	23	Give results of additional analyses, if done (e.g., sensitivity or subgroup analyses, meta-regression [see Item 16]).	-----
**DISCUSSION**			
Summary of evidence	24	Summarize the main findings including the strength of evidence for each main outcome; consider their relevance to key groups (e.g., healthcare providers, users, and policy makers).	11-12
Limitations	25	Discuss limitations at study and outcome level (e.g., risk of bias), and at review-level (e.g., incomplete retrieval of identified research, reporting bias).	14

Conclusions	26	Provide a general interpretation of the results in the context of other evidence, and implications for future research.	14-15
**FUNDING**			
Funding	27	Describe sources of funding for the systematic review and other support (e.g., supply of data); role of funders for the systematic review.	15

Appendix B: Strategies and keywords used for literature search

Set	Query	Results
#1	“influenza vaccines”[mesh] AND (“seasonal” [tiab] OR "seasons"[mesh] OR “seasonal influenza vaccination”[tiab])	1982
#2	“Tropics”[tiab] OR “Asia”[mesh] OR “Africa”[mesh] OR “Pacific”[tiab] OR “Latin America”[mesh] OR “Tropical”[tiab] OR “subtropical”[tiab] OR “Africa South of the Sahara”[mesh] OR “sub-Sahara”[tiab] OR “AFRO”[tw] OR “Algeria”[tw] OR “Angola”[tw] OR “Benin”[tw] OR “Botswana”[tw] OR “Burkina Faso”[tw] OR “Burundi”[tw] OR “Cameroon”[tw] OR “Cabo Verde”[tw] OR “Central African Republic”[tw] OR “Chad”[tw] OR “Côte d'Ivoire”[tw] OR “Congo”[tw] OR “Equatorial Guinea”[tw] OR “Eritrea”[tw] OR “Ethiopia”[tw] OR “Gabon”[tw] OR “Gambia”[tw] OR “Ghana”[tw] OR “Guinea”[tw] OR “Guinea-Bissau”[tw] OR “Kenya”[tw] OR “Liberia”[tw] OR “Madagascar”[tw] OR “Malawi”[tw] OR “Mali”[tw] OR “Mauritania”[tw] OR “Mauritius”[tw] OR “Mozambique”[tw] OR “Namibia”[tw] OR “Niger”[tw] OR “Nigeria”[tw] OR “Rwanda”[tw] OR “Senegal”[tw] OR “Sierra Leone”[tw] OR “South Africa”[tw] OR “Togo”[tw] OR “Uganda”[tw] OR “Tanzania”[tw] OR “Zambia”[tw] OR “Zimbabwe”[tw] OR “EMRO”[tw] OR “Afghanistan”[tw] OR “Israel” [tw] OR “Bahrain”[tw] OR “Djibouti”[tw] OR “Egypt”[tw] OR “Iran”[tw] OR “Iraq”[tw] OR “Jordan”[tw] OR “Kuwait”[tw] OR “Libya”[tw] OR “Morocco”[tw] OR “Oman”[tw] OR “Pakistan”[tw] OR “Qatar”[tw] OR “Saudi Arabia”[tw] OR “Somalia”[tw] OR “Sudan”[tw] OR “Tunisia”[tw] OR “United Arab Emirates”[tw] OR “Yemen”[tw] OR “PAHO”[tw] OR “Anguilla”[tw] OR “Antigua”[tw] OR “Barbuda”[tw] OR “Argentina”[tw] OR “Bahamas”[tw] OR “Barbados”[tw] OR “Belize”[tw] OR “Venezuela”[tw] OR “Bolivia”[tw] OR “Brazil”[tw] OR “Cayman Islands”[tw] OR “Chile”[tw] OR “Colombia”[tw] OR “Costa Rica”[tw] OR “Cuba”[tw] OR “Dominica”[tw] OR “Dominican Republic”[tw] OR “Ecuador”[tw] OR “El Salvador”[tw] OR “Grenada”[tw] OR “Guatemala”[tw] OR “Guyana”[tw] OR “Haiti”[tw] OR “Honduras”[tw] OR “Jamaica”[tw] OR “Mexico”[tw] OR “Montserrat”[tw] OR “Netherland Antilles”[tw] OR “Nicaragua”[tw] OR “Panama”[tw] OR “Paraguay”[tw] OR “Peru”[tw] OR “Saint Kitts and Nevis”[tw] OR “Saint Lucia”[tw] OR “Saint Vincent ”[tw] OR “Grenadines”[tw] OR “Suriname”[tw] OR “Trinidad”[tw] OR “Tobago”[tw] OR “Turks”[tw] OR “Caicos”[tw] OR “Uruguay”[tw] OR “SEARO”[tw] OR “Bangladesh”[tw] OR “Bhutan”[tw] OR “India”[tw] OR “Indonesia”[tw] OR “Maldives”[tw] OR “Myanmar”[tw] OR “Nepal”[tw] OR “Sri Lanka”[tw] OR “Thailand”[tw] OR “Timor-Leste”[tw] OR “WPRO”[tw] OR “Brunei Darussalam”[tw] OR “Cambodia”[tw] OR “China”[tw] OR “Cook Islands”[tw] OR “Fiji”[tw] OR “French Polynesia”[tw] OR “Guam”[tw] OR “Hong Kong”[tw] OR “Kiribati”[tw] OR “Lao”[tw] OR “Malaysia”[tw] OR “Marshall islands”[tw] OR “Nauru”[tw] OR “New Caledonia”[tw] OR “Papua New Guinea”[tw] OR “Philippines”[tw] OR “Samoa”[tw] OR “Singapore”[tw] OR “Solomon islands”[tw] OR “Taiwan”[tw] OR “Tonga”[tw] OR “Vanuatu”[tw] OR “Viet Nam”[tw]	1248012
3	#1 AND #2 AND (“effectiveness”[tiab] OR “efficacy”[tiab])	76
4	#1 AND #2 AND (“timing” [tiab] OR “composition” [tiab])	17
5	#1 AND #2 AND “policy”[tiab]	12
6	#1 AND #2 AND (“campaign”[tiab] OR “coverage”[tiab] OR “uptake”[tiab])	50
7	#1 AND #2 AND (“production” [tiab] OR “availability” [tiab] OR "manufacturing"[tiab] OR "manufacturer"[tiab])	25

Note: [tiab] – title abstract; [tw] – text word; [mesh] – medical subject heading;

Appendix C: List of countries and territories in the tropics and subtropics included in the review

AFRO	Algeria, Angola, Benin, Botswana, Burkina Faso, Burundi, Cabo Verde, Cameroon, Central African Republic, Chad, Congo (the), Côte d'Ivoire, Democratic Republic of the Congo, Equatorial Guinea, Eritrea, Ethiopia, Gabon, Gambia, Ghana, Guinea, Guinea-Bissau, Kenya, Liberia, Madagascar, Malawi, Mali, Mauritania, Mauritius, Mozambique, Namibia, Niger, Nigeria, Rwanda, Senegal, Sierra Leone, South Africa, Togo, Uganda, United Republic of Tanzania, Zambia, Zimbabwe
EMRO	Afghanistan, Bahrain, Djibouti, Egypt, Iran (Islamic Republic of), Iraq, Jordan, Kuwait, Lebanon, Libya, Morocco, Oman, Pakistan, Qatar, Saudi Arabia, Somalia, Sudan, Syrian Arab Republic, Tunisia, United Arab Emirates, Yemen
PAHO	Anguilla, Antigua and Barbuda, Argentina, Bahamas, Barbados, Belize, Bolivia (Plurinational State of), Brazil, Cayman Islands, Chile, Colombia, Costa Rica, Cuba, Dominica, Dominican Republic, Ecuador, El Salvador, Grenada, Guatemala, Guyana, Haiti, Honduras, Jamaica, Mexico, Montserrat, Netherland Antilles, Nicaragua, Panama, Paraguay, Peru, Saint Kitts and Nevis, Saint Lucia, Saint Vincent and the Grenadines, Suriname, Trinidad and Tobago, Turks and Caicos Islands, Uruguay, Venezuela (Bolivarian Republic of)
SEARO	Bangladesh, Bhutan, India, Indonesia, Maldives, Myanmar, Nepal, Sri Lanka, Thailand, Timor-Leste
WPRO	American Samoa, Brunei Darussalam, Cambodia, China, China, Hong Kong SAR, China – Province of Taiwan, Cook islands, Democratic People’s Republic of Korea, Fiji, French Polynesia, Guam, Kiribati, Lao People's Democratic Republic, Malaysia, Marshall islands, Nauru, New Caledonia, Niue, Palau, Papua New Guinea, Philippines, Samoa, Singapore, Solomon islands, Tonga, Vanuatu, Viet Nam
EURO	Israel

Appendix D: Potential risk of bias (shaded in grey) in cohort vaccine effectiveness studies. Risk of bias assessed using Newcastle-Ottawa Scale

		Potential risk of bias in							
**Author (Year)**	**Reference**	**Selection of exposed cohort**	**Selection of non-exposed cohort**	**Exposure ascertainment**	**Outcome not present at start of study**	**Comparability of exposed and non-exposed cohort**	**Outcome ascertainment**	**Follow up not long enough**	**Loss to follow up**
Chan (2013)	[121]	No	No	No	No	No	No	No	No
Gurfinkel (2004)	[140]	No	No	Yes	No	Yes	Yes	No	No
Gutierrez (2001)	[90]	Yes	No	No	No	Yes	No	No	Yes
Hung (2010)	[104]	No	No	No	No	No	No	No	No
Kheok (2008)	[145]	Yes	No	No	Yes	Yes	Yes	No	Yes
Kittikraisak (2014)	[123]	No	No	No	No	Yes	No	No	Yes
Menon (2008)	[136]	Yes	No	No	Yes	No	Yes	No	No
Morales (2004)	[127]	Yes	No	Yes	No	Yes	Yes	No	No
Qureshi (2000)	[146]	No	No	No	Yes	No	Yes	No	No
Samad (2006)	[126]	Yes	No	No	No	Yes	Yes	No	No
Stamboulian (1999)	[80]	Yes	Yes	No	No	No	No	No	Yes
Wang (2007)	[116]	No	No	No	No	No	No	No	Yes

Appendix E: Potential risk of bias (shaded in grey) in case control vaccine effectiveness studies – Risk of bias assessed using Newcastle-Ottawa Scale

		Potential risk of bias in							
**Author (Year)**	**Reference**	**Case selection**	**Case representativeness**	**Control representativeness**	**Control selection**	**Comparability of case and control**	**Exposure ascertainment**	**Exposure ascertainment different for control**	**Non-response rate**
Dawood (2014)	[115]	No	No	No	Yes	No	No	No	Yes
Fu (2013)<br>He (2013)<br>Yang (2012)	[54]<br>[53]<br>[55]	Yes	No	No	No	No	No	No	Yes
Ho (2014)	[44]	Yes	No	No	No	No	No	No	Yes
Mustafa (2003)	[147]	No	No	No	No	No	No	No	Yes
Van Vuuren (2009)	[113]	Yes	No	Yes	No	No	No	No	No

Appendix F: Potential risk of bias in randomized controlled trials for vaccine efficacy studies – Risk of bias assessed using Cochrance risk of bias assessment tool. High risk of potential bias or lack of information to assess risk of bias is shaded in grey.

		Potential risk of bias in						
**Author (Year)**	**Refer ence**	**Allocation sequence generation**	**Allocation concealment**	**Blinding of participants**	**Blinding of investigator**	**Blinding of outcome assessor**	**Completeness of outcome data**	**Outcome reporting**
Bracco Neto (2009)	[49]	Low	Not known	Low	Not known	Low	Not known	Low
Cowling (2010)	[47]	Low	Low	Low	Low	Low	Low	Low
De Villiers (2009)	[43]	Low	Not known	Low	Low	Low	Low	Low
Forrest (2011)	[98]	Low	High	High	High	High	Low	Low
Isahak (2007)	[99]	High	High	Low	High	High	Low	Low
Jain (2013)	[48]	Low	Low	Low	Low	Low	Low	Low
Jianping (1999)	[101]	High	High	High	High	High	Not known	High
Lum (2010)	[51]	Low	Low	Low	Low	Low	Low	Low
Madhi (2011)	[143]	Low	High	Low	Low	Low	Low	Low
Madhi (2014)	[58]	Low	Low	Low	Low	Low	Low	Low
Phrommintikul (2011)	[139]	Low	High	High	High	Low	Low	Low
Plasai (2006)	[100]	High	High	High	High	High	Low	High
Praditsuwan (2005)	[42]	Low	High	Low	High	Low	Low	Low
Tam (2007)	[50]	Low	Low	Low	Low	Low	Low	Low
Wongsurakiat (2004)	[137]	Low	High	Low	High	Low	Low	Low
Zaman (2008)	[57]	Low	Low	Low	Not known	Low	Low	Low

Note: Kositanont et al (2004) was not assessed for risk of bias as we were not able to retrieve the full text of the paper

Appendix G: Seasonal influenza vaccine coverage in the tropics and subtropics

Author (Year)	Reference	Country	Period	Vaccinated group	Sample size	Coverage	Remarks
Bellei (2007)	[95]	Brazil	2001-03	Health professionals	203	19.7%	
Cabral (2006)	[82]	Brazil	2004	>60y		38.3 – 50.2%	
Cesar (2008)	[83]	Brazil	2005	>60y		80.2 – 88.1%	
Chan (2013)	[97]	Hong Kong SAR	2012	Health professionals	1300	55.9%	

Author (Year)	Reference	Country	Period	Vaccinated group	Sample size	Coverage	Remarks
De Lataillade (2009)	[77]	Argentina, Chile, China, Republic of Korea, South Africa, China (Taiwan)	2005-06	All ages	>=18y: 17047<br><18y: 7417	**Overall:**<br>Argentina: 14%<br>Chile: 25%<br>China (Taiwan): 17%<br>Republic of Korea: 41%<br>China: 11%<br>South Africa: 13%<br>**Children:**<br>China, Chile, China (Taiwan), Republic of Korea: 23 – 62%<br>Argentina: 10%<br>**Adults:**<br>Argentina: 10%<br>Chile: 21%<br>China (Taiwan): 8%<br>Republic of Korea: 26%<br>China: 4%<br>South Africa: 13%<br>**Elderly:**<br>Argentina: 38%<br>Chile: 70%<br>China (Taiwan): 42%<br>Republic of Korea: 75%<br>China: 4%<br>South Africa: 13%	China, South Africa (only urban);<br><br>Public funded: Argentina, Chile, China (Taiwan) – Province of China, Republic of Korea;<br>User paid: China, South Africa
Donalisa (2006)	[84]	Brazil	2003	>60y		63.2%	
Francisco (2006)	[85]	Brazil	2001-02	>60y		62 – 67.6%	

Author (Year)	Reference	Country	Period	Vaccinated group	Sample size	Coverage	Remarks
Geronutti (2008)	[89]	Brazil	2006	>60y		83.8%	Clinic attendees
Gutierrez (2001)	[90]	Brazil	1999	>60y		70.1%	Hospital attendees
Koul (2014)	[92]	India	2012-13	Pregnant women	1000	0%	
Lau (2010)	[93]	Hong Kong SAR	2005-06	Pregnant women	568	3.9%	
Lee (2013)	[78]	China (Taiwan)	2011-12	All ages	2,582,859 doses	Elderly >65y: 40.2%<br>Pre-school children: 31.9%<br>Grade 1 – 4: 72.2%<br>High risk (grade 5) to <65y: 41.2%	
Lima-Costa (2008)	[88]	Brazil	2003	>60y		66.3%	
Owusu (2013)	[79]	Thailand	2010-12	All ages	> 8million doses	Elderly: 11.8%<br>Young children: 1.5%<br>Pregnant women: <1%	

Author (Year)	Reference	Country	Period	Vaccinated group	Sample size	Coverage	Remarks
Ropero-Alvarez (2009)	[26]	Latin America and the Caribbean	2006-08	Elderly		Argentina (>65y): 50%<br>Bermuda (>60y): 60 – 65%<br>Brazil (>60y): 85.7 – 86.9%<br>Chile (>60y): 88.6 – 89.1%<br>Cuba (>65y): 100%<br>Ecuador (>65y): 57.5 – 67%<br>El Salvador (>60y): 92 – 100%<br>Guatemala (>60y): 100%<br>Honduras (>60y): 83 – 90%<br>Mexico (>60y): 84.9 – 93.3%<br>Panama (>60y): 79 – 86.2%<br>Paraguay (>60y): 73 – 74%<br>Uruguay (>65y): 29.2 – 31.1%<br>Venezuela (>60y): 20.6 – 61.9%	
Santos (2009)	[91]	Brazil	1999	>60y		76.5%	Hospital attendees
Stamboulian (1999)	[80]	Argentina	1993-97	>65y	> 3.7million doses	5.9 – 29.5%	
Takayanagi (2007)	[96]	Brazil	2002	Health professionals		34.4%	
Yuet (2013)	[94]	Hong Kong SAR	2010-11	Pregnant women	2822	1.7% (95% CI: 1.3 – 2.3%)	

Appendix H: Seasonal influenza effectiveness in the elderly

Author (Year) [Reference]	Study type, year, place	ILI	Laboratory-confirmed influenza	Pneumonia	Hospitalization	Mortality	Remarks
Breteler (2013) [18]	Meta-analysis	LAIV: 4% (ns); TIV: 59% (44 – 70)	LAIV: 43% (25 – 56); TIV: 58% (23 – 78)		All-cause (LAIV): 8% (ns); P&I (LAIV): no effect; P&I (TIV): 26% (ns)		RCTs (7), cohort (2), case control (1) 9/10 studies from tropics Low middle income countries
Darvishian (2014) [62]	Meta-analysis	36% (ns)	77% (ns)		P&I: 25% (6 – 40)	All-cause: 36% (8 – 56)	1/14 studies from tropics (China - Province of Taiwan)
Beyer (2013) [61]	Meta-analysis	~40%	~50%	~30%		P&I: ~30%	Jefferson 2010 review reanalysed
Jefferson (2010) [102]	Meta-analysis			Healthy elderly: 41%;	Healthy elderly: 50%; At risk elderly: 26%	P&I: 8%; All-cause: 61%	1/75 studies from tropics (China, Hong Kong SAR)
Moreno (2009) [105]	Meta-analysis				20% – 26%		1/28 studies from tropics (China - Province of Taiwan)
Rivetti (2006) [106]	Meta-analysis				P&I: 27% (21 – 33) Respiratory: 22% (15 – 28) CVD: 24% (18 – 30)	All-cause: 47% (39 – 54)	Review updated by Jefferson in 2010; 0/64 studies from tropics
Vu (2002) [107]	Meta-analysis				P&I: 33% (27 – 38) Respiratory: 30% (25 – 35)	All-cause: 50% (45 – 56)	
Gross (1995) [103]	Meta-analysis	ARI: 56% (39 – 68)		53% (35 – 66)	50% (28 – 65)	All-cause: 68% (56 – 76)	0/20 studies from tropics

Author (Year) [Reference]	Study type, year, place	ILI	Laboratory-confirmed influenza	Pneumonia	Hospitalization	Mortality	Remarks
**Studies from the tropics**							
Forrest (2011) [98]	RCT (2002) South Africa	inconclusive					LAIV v/s TIV
Gutierrez (2001) [90]	Cohort (2000) Brazil	6% (ns)					TIV
Isahak (2007) [99]	Non-RCT (2003-04) Malaysia	55% to 76%					TIV v/s placebo
Plasai (2006) [100]	Non-RCT (2004-05) Thailand	48%					TIV v/s no vaccine
Jianping (1999) [101]	RCT (1996-97) China	74%					TIV<br>Control group unclear
Praditsuwan (2005) [42]	RCT Thailand	56% (14 – 77)	Significant reduction				TIV v/s placebo<br>No effect on reduction of serious complications
Façanha (2005) [108]	Cohort (1995-2003) Brazil				No effect	No effect	

**Author (Year) [Reference]**	**Study type, year, place**	**ILI**	**Laboratory-confirmed influenza**	**Pneumonia**	**Hospitalization**	**Mortality**	**Remarks**
Brondi (2000) [109] Daufenbach (2009) [110] Ferrer (2008) [111] Francisco (2005) [120]	Ecological (1998-2002) Brazil				Modest reduction		Articles in Portuguese – not retrieved; Identified through cross-references
De Villiers (2009) [43]	RCT (2001) South Africa		Good match: 42% (23 – 57); Any match: 42% (22 – 57);		P&I: Inconclusive	Inconclusive	LAIV v/s placebo
Hung (2010) [104]	Cohort (2008-09) China, Hong Kong SAR			43% (36 – 49)	CVD: 41% (21 – 56); ICU: 55% (6 – 78); Stroke: 33%	All-cause: 35% (23 – 45)	TIV v/s PPV[1] v/s TIV+PPV v/s placebo; VE against myocardial infarction (48%)
Van Vuuren (2009) [113]	Case control (2003-04) South Africa				CVD: 15% (ns); Respiratory: 15% (ns)	All-cause: 24% (1 – 41)	TIV

Author (Year) [Reference]	Study type, year, place	ILI	Laboratory-confirmed influenza	Pneumonia	Hospitalization	Mortality	Remarks
REVELAC-i (2013) [114]	Case control (2013) Central, South America				Influenza:<br>Chile: 45% (7 – 65);<br>Brazil: 77% (62 – 86);<br>Argentina, Colombia, Costa Rica, El Salvador, Honduras, Panama, Paraguay: 57% (43 – 68)		TIV
Dawood (2014) [115]	Case control (2010-11) Thailand				Influenza: 47% (5 – 71)		TIV<br>Vaccination coverage low;<br>Vaccine and virus strain match good
Stamboulian (1999) [80]	Cohort (1993) Argentina				P&I: 38% (21 – 51);<br>P&I (elderly high risk): 45% (29 – 58);		TIV<br>Vaccination coverage: 6 – 30%
Wang (2007) [116]	Cohort (2001) China - Province of Taiwan				Overall: 11% (8 – 14)<br>***high risk elderly:***<br>All-cause: 20% (16 – 23)<br>Stroke: 4% (ns)<br>CVD: 9% (ns)<br>P&I: 31% (17 – 42)<br>***Low risk elderly:***<br>All-cause: 33% (19 – 26)<br>Stroke: 36% (24 – 46)<br>CVD: 16% (2 – 29)<br>P&I: 33% (18 – 47)	All-cause: 44% (40 – 48);<br>Pneumonia: 53% (35 – 66);<br>Stroke: 65% (55 – 73);<br>CVD: 22% (4 – 36);	TIV<br>VE against mortality from renal disease (60%), diabetes (55%), COPD (45%)
Yung (2013) [118]	Cohort (2010-12) Singapore				All-cause emergency: 11% (7 – 15)	75% (70 – 80)	TIV<br>All ages, all cause emergency hospitalizations

Author (Year) [Reference]	Study type, year, place	ILI	Laboratory-confirmed influenza	Pneumonia	Hospitalization	Mortality	Remarks
Antunes (2007) [119]	Ecological (1998-2002) Brazil					P&I: 26%	TIV<br>P&I mortality reduced by 26% after vaccination campaign (1998-2002) compared to before vaccination (1993-97)
Francisco (2005) [120]	Ecological (1980-2000) Brazil					All-cause (men): 7%; All-cause (women): 6%	TIV<br>VE (1998-2000) after vaccination compared to before vaccination (1993-97)
Chan (2013) [121]	Cohort (2010-11) China, Hong Kong SAR					All-cause: 28% (5 – 46); P&I: 20% (2 – 38)	TIV

Note: [1] PPV – Pneumococcal Polysaccharide Vaccine
(ns) – Not statistically significant at the 5% significance level

Appendix I: Seasonal influenza effectiveness in children

**Author (Year) [Reference]**	**Study type, year, place**	**ILI**	**Laboratory confirmed Influenza**	**Remarks**
Breteler (2013) [18]	Meta-analysis<br>Low middle income countries	LAIV: 36% (28 - 54);<br>TIV: 27% (21 - 33)	LAIV (good match): 72% (65 - 77);<br>TIV (good match): 81% (58 - 92)	RCTs (10), cohorts (2)<br>6/12 studies from tropics;<br>LAIV (P&I hospitalization): no effect
Luksic (2013) [63]	Meta-analysis	LAIV: 31% (25 – 40);<br>TIV: 32% (20 – 53)	**LAIV:**<br>Good match: 82% (77 - 89);<br>Any match: 77% (69 – 86);<br>**TIV:**<br>Any match: 67% (58 - 78)	**LAIV:**<br>RCTs (12), cohorts (4)<br>2/16 studies from tropics<br>**TIV:**<br>RCTs (11), cohorts (5), case control (2)<br>1/18 studies from tropics
Tricco (2013) [64]	Meta-analysis (1970-2009)		**LAIV:**<br>Good match: 77% (67 - 86);<br>Poor match: 60% (44 – 71);<br>**TIV:**<br>Good match: 65% (57 – 72);<br>Poor match: 56% (43 – 66);	RCTs (34)<br>5/34 studies from tropics<br>VE against mismatched influenza A (LAIV-75%, TIV-62%) significantly higher than mismatched influenza B (LAIV-42%, TIV-52%) respectively
Osterholm (2012) [65]	Meta-analysis (1967-2011)		LAIV: 83% (69 – 91)	RCTs (6);<br>3/6 studies from tropics
DiazGranados (2012) [66]	Meta-analysis		**LAIV:**<br>Any match: 80% (70 – 87)<br>Good match: 88% (83 – 92);<br>Poor match: 80% (50 – 92);<br>**TIV:**<br>Any match: 48% (31 – 61)<br>Good match: 48% (15 – 68);<br>Poor match: 49% (3 – 73);	RCTs or CCTs (30)<br>2/30 studies from tropics (Cuba, multi-site, Asia);<br>LAIV efficacy better than TIV in children

Author (Year) [Reference]	Study type, year, place	ILI	Laboratory confirmed Influenza	Remarks
Michiels (2011) [67]	Meta-analysis	36% (24 – 46)	69% (55 – 78);	RCTs (3), CCT (1);<br>0/4 studies from tropics;
Carter (2011) [68]	Meta-analysis		**LAIV v/s placebo:**<br>Good match:69%(53-80);<br>Poor match:87%(77-93);<br>Otitis-85%(78-90)<br>**LAIV v/s TIV:**<br>Good match:53%(27-69);<br>Poor match:54%(42-65);<br>Otitis-54%(27-72)	RCTs (8);<br>5/8 studies from tropics;<br>LAIV more effective than TIV in children with recurrent ARI and adolescents with asthma<br>**LAIV v/s placebo:**<br>Otitis-85%(78-90)<br>**LAIV v/s TIV:**<br>Otitis-54%(27-72)
Rhorer (2009) [69]	Meta-analysis (LAIV)		Good match (2 doses): 75% (71 - 79);<br>Good match (1 dose): 58% (49 - 66);	RCTs (9);<br>4/6 LAIV-placebo studies from tropics;<br>1/3 LAIV-TIV studies from tropics;<br>Good match (H1N1): 85% (78 - 90);<br>Good match (H3N2): 76% (70 - 81);<br>Good match (B): 73% (63 - 80);<br>LAIV more effective than TIV in children
Jefferson (2008) [70]	Meta-analysis	LAIV: 33% (28 – 38);<br>TIV: 36% (24 – 46)	LAIV: 82% (71 – 89);<br>TIV: 59% (41 – 71)	RCTs (17), cohorts (19), case control (11);<br>2/47 studies from tropics;
Manzoli (2007) [71]	Meta-analysis	Overall: 36% (31 – 40);<br>LAIV: 35% (30 – 40);<br>TIV: 45% (33 – 55);<br>Good match: 41% (31 – 49);<br>Poor match: 32% (27 – 36);	Overall: 67% (51 – 78);<br>LAIV: 72% (38 – 87);<br>TIV: 62% (45 – 75);<br>Good match: 61% (40 – 75);<br>Poor match: 78% (63 – 87);	RCTs (19);<br>1/19 studies from tropics;<br>**Otitis Media:**<br>Overall: 51% (21 – 70);<br>LAIV: 78% (25 – 90);<br>TIV: 32% (ns);

Author (Year) [Reference]	Study type, year, place	ILI	Laboratory confirmed Influenza	Remarks
Negri (2005) [72]	Meta-analysis (1985-2001)	Overall: 33% (29 – 36);<br>LAIV: 34% (3 – 38);<br>TIV: 33% (22 – 42);	Overall: 74% (57 – 84);<br>LAIV: 80% (53 – 91);<br>TIV: 65% (45 – 77);	RCTs (13);<br>1/13 studies from tropics
Ruben (2004) [73]	Review		Influenza A: 31% - 91%;<br>Influenza B: 45%;	RCTs (7);<br>0/7 studies from tropics;<br>Otitis Media: 32% - 36%
**Studies from the tropics**				
Jianping (1999) [101]	RCT (1996-97) China	85%		TIV<br>Control group unclear
Cowling (2010) [47]	RCT (2009) China, Hong Kong SAR	8% (ns)	A(H1N1): 25% (ns);<br>A(H3N2): 50% (ns);<br>B: no effect	TIV v/s placebo
Jain (2013) [48]	RCT (2010-11) Bangladesh, Dominican Republic, Honduras, Lebanon, Panama, Philippines, Thailand		Good match: 48% (16% - 67%);<br>Any match: 59% (41% - 72%);<br>Influenza A: 57% (36 – 71);<br>Influenza B: 50% (17 – 70);<br>Any severity: 55% (39 - 67);<br>Mod to severe: 73% (47 - 86);<br>3 – 4 y: 35% (ns);<br>5 – 8 y: 68% (50 - 79)	QIV v/s hepatitis A vaccine
Bracco Neto (2009) [49]	RCT (2001-02) Argentina, Brazil, South Africa		LAIV (1 dose) any match: 56% (43 – 67);<br>LAIV (1 dose) good match: 58% (45 – 68);<br>LAIV (2 doses) any match: 72% (62 – 80);<br>LAIV (2 doses) good match: 74% (64 – 81);	LAIV<br>Two doses provided additional protection; protection persisted into 2nd year without revaccination

**Author (Year) [Reference]**	**Study type, year, place**	**ILI**	**Laboratory confirmed Influenza**	**Remarks**
Tam (2007) [50]	RCT (2000-03) China, China, Hong Kong SAR, India, Malaysia, Philippines, Singapore, China - Province of Taiwan, Thailand		LAIV good match: 70% (61 – 77);<br>LAIV any match: 68% (59 – 75)	LAIV v/s placebo
Lum (2010) [51]	RCT (2002-03) Bangladesh, China, Hong Kong SAR, Malaysia, Mexico, Philippines, Singapore, Thailand		Good match (LAIV): 78% (51 – 91);<br>Any match (LAIV): 64% (36 – 80)	LAIV v/s placebo
Belshe (2007) [52]	RCT (2004-05) Asia (3), Middle East		Good match: 45% (22% - 61%);<br>Poor match: 58% (47% - 67%);<br>Any match: 55% (45% - 63%);	LAIV v/s TIV<br>Subjects from 3 countries in Asia (6%), United States (49%), Europe, Middle East (45%)
He (2013) [53]<br>Fu (2013) [54]<br>Yang (2012) [55]	Case control (2009-12) China		2010-12: 34% (5 – 54);<br>6mo-3y (2 doses): 47% (9 – 70);<br>6mo-3y (1 dose): 5% (ns)<br><br>2010 (2 doses): 58% (44 – 68);<br>2010 (1 dose): 33% (16 – 46);<br>2009 (2 doses): 52% (41 – 60);<br>2009 (1 dose): 32% (19 – 43)	TIV<br>Partial vaccination had no protection;<br>Till 6mo: 35% (5% - 55%);<br>After 6mo till 1y: no protection

**Author (Year) [Reference]**	**Study type, year, place**	**ILI**	**Laboratory confirmed Influenza**	**Remarks**
REVELAC-i (2013) [114]	Case control (2013) Central, South America		Brazil: 20%; Chile: 75% (32 – 98); Colombia: 77%; Costa Rica, El Salvador, Honduras, Panama: 48% (22 – 66)	TIV
Victor (2013) [122]	RCT (2009-10) Senegal		33% (6 – 52); Contacts: 19% (ns)	TIV v/s inactivated polio vaccine
Kittikraisak (2014) [123]	Cohort (2011-13) Thailand		2011-12: 62% (0 – 91); 2012-13: 37% (0 – 76)	TIV
Gattas (2013) [56]	RCT (2009) Brazil		Family contacts: 61% (6 – 85)	TIV v/s meningococcal and varicella vaccine Effectiveness of vaccination of children against influenza in household contacts

Note: (ns) – Not statistically significant at the 5% significance level

Appendix J: Seasonal influenza vaccine effectiveness in healthy adults

Author (Year) [Reference]	Study type, year, place	ILI	Laboratory confirmed Influenza	Remarks
Breteler (2013) [18]	Meta-analysis Low middle income countries	62% (45 – 73);<br>ARI: 75% (ns)	**TIV:**<br>82% (61 – 92)	RCTs (5), cohorts (5)<br>5/10 studies from tropics
Tricco (2013) [64]	Meta-analysis (1970-2009)		**TIV:**<br>Good match: 65% (54 – 73);<br>Poor match: 52% (37 – 63);<br>Poor match (influenza A): 64% (23 – 82);<br>Poor match (influenza B): 52% (19 – 72)	RCTs (34)<br>5/34 studies from tropics
DiazGranados (2012) [66]	Meta-analysis		**LAIV:**<br>Any match: 39% (16 – 55)<br>Good match: 8% (ns);<br>Poor match: 53% (15 – 74);<br>**TIV:**<br>Any match: 59% (50 – 66)<br>Good match: 57% (43 – 68);<br>Poor match: 50% (22 – 68);	RCTs or CCTs (14)<br>2/14 studies from tropics;<br>LAIV efficacy better than TIV in children
Osterholm (2012) [65]	Meta-analysis (1967-2011)	**TIV:** 69% (60 – 93)	**TIV:** 59% (51 – 67)	RCTs (11);<br>1/11 studies from tropics

Author (Year) [Reference]	Study type, year, place	ILI	Laboratory confirmed Influenza	Remarks
Jefferson (2010) [124]	Meta-analysis	**LAIV:**<br>Any match: 10% (4 – 16)<br>Good match: 8% (ns);<br>Poor match: 11% (3 – 18);<br>**TIV:**<br>Any match: 20% (11 – 29)<br>Good match: 30% (17 – 41);<br>Poor match: 7% (ns);	**LAIV:**<br>Any match: 62% (45 – 73)<br>Good match: 56% (19 – 76);<br>Poor match: 64% (18 – 84);<br>**TIV:**<br>Any match: 61% (48 – 70)<br>Good match: 73% (54 – 84);<br>Poor match: 44% (23 – 59);	RCTs (35)<br>2/35 studies from tropics;<br>**LAIV:**<br>Pneumonia: 75% (ns)<br>**TIV:**<br>Pneumonia: 20% (ns)<br>Hospitalization: 11% (ns)
Demicheli (2014) [74]	Meta-analysis	**TIV:** 30% (17 – 41)	**TIV:**<br>Good match: 80% (56 – 91);<br>Poor match: 50% (27 – 65)	RCTs (38), CCTs (8);<br>2/46 studies from tropics;
**Studies from the tropics**				
Jianping (1999) [101]	RCT	39%		TIV<br>Control unclear
Hui (2008) [125]	Cohort (2008) Malaysia	53%		TIV<br>Dental students and faculty
Samad (2006) [126]	Cohort (2001) Malaysia	73%		TIV<br>Factory workers
Morales (2004) [127]	Cohort (2000-01) Colombia	63%		TIV<br>Bank employees
Ho (2014) [44]	Case control (2010-13) Singapore		A(H1N1)pdm: 84% (78 – 88);<br>A(H3N2): 33% (4 – 57);<br>B: 84% (79 – 86)	TIV

Author (Year) [Reference]	Study type, year, place	ILI	Laboratory confirmed Influenza	Remarks
Ntshoe (2014) [45]	Case control (2005-09) South Africa		2005: 49% (5 – 73);<br>2006: no effect;<br>2007: 12% (ns);<br>2008: 67% (12 – 90);<br>2009: no effect	TIV<br>All ages
McAnerny (2013) [46]	Case control (2010-12) South Africa		2010: 58% (7 – 81);<br>2011: 59% (22 – 78);<br>2012: 50% (40 – 85)	TIV<br>All ages

Note: (ns) – Not statistically significant at the 5% significance level

Appendix K: Seasonal influenza vaccine effectiveness in pregnant women

Author (Year) [Reference]	Study type, year, place	ILI or laboratory-confirmed influenza	ILI or laboratory-confirmed influenza	Hospitalization	Other infant outcomes	Remarks
Steinhoff (2014) [134]	Review				**Preterm:** 16% (ns), 25%, 73%; **SGA:** 7% (ns), 25%, 71%; **LBW:** 27% (5 -44)	RCTs (8); 4/8 studies on pandemic vaccine; 1/4 studies on TIV from tropics;
Omer (2012) [129] Blanchard-Rohner (2011) [130] Skowronski (2009) [131] Naleway (2006) [132]	Review	ILI: no effect, 44% (9 – 65), 36%; Ab titres: high titres , similar	ARI: no effect, 41% – 63%; Ab titres: higher	No effect, 39%, 45% – 48%, 92%; Ab titres: increased	**Preterm:** 40% (6 – 62); During peak: 72% (26 – 89); **SGA:** 69% (25 – 87) ;	RCTs (2), cohorts (10), Case control (1); 1/13 studies from tropics; Most studies did not use laboratory-confirmed influenza as outcomes
Galvao (2013) [128]	Review	ILI: Significant reduction	Significant reduction; ARI: no effect		Preterm, SGA, Still birth, neonatal mortality: no effect	RCT (1), cohorts (7); 1/8 studies from tropics
Mak (2008) [133]	Review	ARI: no effect (United States studies)	61% (9 – 84); ILI: 65%; ARI: no effect (United States studies)			1/8 studies from tropics;
**Studies from the tropics**						

**Author (Year) [Reference]**	**Study type, year, place**	**ILI or laboratory-confirmed influenza**	**ILI or laboratory-confirmed influenza**	**Hospitalization**	**Other infant outcomes**	**Remarks**
Madhi (2014) [58]	RCT (2011-12) South Africa	**HIV infected mothers:** LCI: 58% (0.2 – 81); ILI: no effect **HIV non-infected mothers:** LCI: 50% (15 – 71); ILI: no effect	**HIV infected mothers:** LCI: 27% (ns); ILI: no effect **HIV non-infected mothers:** LCI: 49% (12 – 70); ILI: no effect			TIV
Zaman (2008) [57]	RCT 2004-05 Banglades h	ILI: 36% (4 – 57);	ILI: 29% (7 – 46); LCI: 63% (5 – 85);		**Preterm:** 28% (ns); **SGA:** 37% (ns)	TIV v/s PPV

Note: PPV: Pneumococcal Polysaccharide Vaccine; LCI: Laboratory confirmed influenza
(ns) – Not statistically significant at the 5% significance level

Appendix L: Seasonal influenza vaccine effectiveness in high risk individuals

Author (Year) [Reference]	Study type, year, place	ILI	laboratory-confirmed influenza	Hospitalization	Mortality	Remarks
**COPD patients**						
Poole (2006) [135]	Meta-analysis	ARI: 11% (ns)	81% (52 – 93)	67% (ns)	No effect	RCTs (11); 1/11 studies from tropics
**Studies from the tropics**						
Kositanont (2004) [59]	RCT (1997-98) Thailand		71%			TIV v/s placebo COPD patients
Menon (2008) [136]	Cohort (2004-06) India	ARI: 67% (p=.005); Mild COPD: 60% (ns); Mod. COPD: 60% (ns); Severe COPD: 75% (p=.02)		ARI: 72% (p=.02)		TIV COPD patients
Wongsurakait (2004) [137]	RCT (1997-98) Thailand	Mild COPD: 84%; Mod. COPD: 45%; Severe COPD: 85%				TIV v/s placebo COPD patients
**Coronary heart disease patients**						
Keller (2008) [138]	Meta-analysis				Coronary Heart Disease: 61% (23 – 80)	RCTs (3); 1/3 studies from tropics; Acute MI: 15% (ns)
**Studies from the tropics**						
Phrommintikul (2011) [139]	RCT (2007-08) Thailand			Acute coronary: 32% (2 – 53); Heart failure: 38% (ns)	38% (ns)	TIV v/s no vaccine CVD patients
Gurfinkel (2004) [140]	Cohort (2001) Argentina				Coronary Heart Disease: 66% (29 – 83);	TIV MI patients; Ischemic events: 41% (14 – 96)

Author (Year) [Reference]	Study type, year, place	ILI	laboratory-confirmed influenza	Hospitalization	Mortality	Remarks
**HIV patients**						
Anema (2008) [141]	Meta-analysis		34% (18 – 64)			RCTs (3), Case control (1); 0/4 studies from tropics
Atashili (2006) [142]	Meta-analysis		27% - 78%			RCT (1), non-RCT (2), outbreak investigation (1); 0/4 studies from tropics
**Studies from the tropics**						
Madhi (2011) [143]	RCT (2011) South Africa	8% (ns); ARI: 16% (ns)	76% (9 – 96)			TIV v/s placebo
**Healthcare professionals**						
Thomas (2013) [144]	Meta-analysis		No effect on those who receive care from HWs	Respiratory: no effect	Respiratory: no effect	RCTs (3); 0/3 studies from tropics
**Studies from the tropics**						
Kheok (2008) [145]	Cohort (2004-05) Singapore	Good match: 51% (34 – 63); Poor match: no effect				TIV
**Pilgrims**						
Breteler (2013) [18]	Meta-analysis Low middle income countries	72% (59 – 80); ARI: 55% (ns)				Cohorts (2); 2/2 from tropics
**Studies from the tropics**						
Qureshi (2000) [146]	Cohort (1999) Pakistan	38% (29 – 45)				TIV Haj pilgrims

Author (Year) [Reference]	Study type, year, place	ILI	laboratory-confirmed influenza	Hospitalization	Mortality	Remarks
Mustafa (2003) [147]	Case control (2000) Malaysia	77% (69 – 83)				TIV<br>Haj pilgrims

Note: (ns) – Not statistically significant at the 5% significance level
